# Inflammatory linear verrucous epidermal nevus in a patient with diabetes

**DOI:** 10.11604/pamj.2022.41.139.33468

**Published:** 2022-02-17

**Authors:** Yosra Htira, Faika Ben Mami

**Affiliations:** 1 Institut National de Nutrition et de Technologies Alimentaires, Tunis, Tunisie

**Keywords:** Epidermal nevus, inflammatory linear verrucous epidermal nevus, frustrating treatments

## Image in medicine

We present the case of a 43-year-old man with diabetes who had erythematous and eczematous oozing plaques over the right leg. There was no family history of similar complaints. The lesions appeared on the early childhood. He was initially treated with a topical corticosteroid-antibiotic combination and antihistamine for a diagnosis of atopic dermatitis. He had a partial response with reduction of oozing, erythema, and crusting, but the lesions persisted. Hair, trunk, nails, oral mucosae, and other organ systems were normal. Skin biopsy specimens from lesions over the leg showed psoriasiform hyperplasia of the epidermis with alternating areas of parakeratosis and hyperkeratosis, acanthosis, spongiosis, and upper dermal lymphocytic infiltrate. Based on the clinical and histologic features, the diagnosis of inflammatory linear verrucous epidermal nevus (ILVEN) was made.

**Figure 1:**
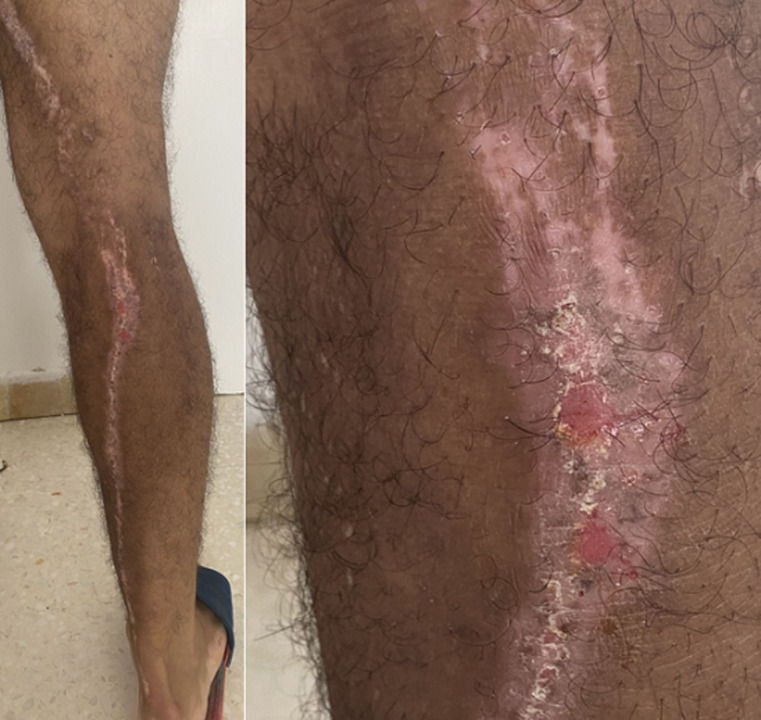
inflammatory linear verrucous epidermal nevus

